# Intersections of Personal Assistance Services for Rural Disabled People and Home Care Workers' Rights

**DOI:** 10.3389/fresc.2022.876038

**Published:** 2022-04-25

**Authors:** Rayna Sage, Krys Standley, Genna M. Mashinchi

**Affiliations:** The Rural Institute for Inclusive Communities, University of Montana, Missoula, MT, United States

**Keywords:** disability, home care workers, policy, home-based services, community-based services

## Abstract

It is very difficult to find and keep workers to provide home-based care for disabled people, especially in rural places. There is a tension between the rights of disabled people and the rights of home-based personal care workers. In this brief review, we explore the intersections of historical and social forces that shaped federal-level policies for both disability rights and the rights of personal care workers, as well as the current state of the policies. This paper provides a narrow focus on federal policies relevant to both groups, while also considering how the urbancentric nature of advocacy and policymaking has failed to address important issues experienced by rural people. In addition to briefly reviewing relevant federal policies, we also explore sources of support and resistance and how urbanormativity, ableism, and sexism intersect to influence how the needs of people with disabilities and their personal care workers are conceptualized and addressed. We conclude with recommendations for how to better address the needs of rural people with disabilities using home-based personal care services and the workers who provide them.

## Introduction

Personal Assistance Services, as part of Home and Community-Based Services funded through Medicaid, are critical for disabled people^1^ to live, work, and recreate in their homes and communities (1). As part of these services, personal care attendants (PCAs—also referred to as personal care aides, personal attendants, and personal assistance service workers) come to disabled peoples' homes to assist them with tasks of daily living such as getting in and out of bed, toileting, meal preparation, housekeeping, transportation, and running errands. Distinct from home healthcare workers, who provide skilled nursing care, PCAs provide more basic care and, in most cases, are not required to have formal training. These services are clearly vital for the wellbeing of people with disabilities. Despite personal assistance services being among the fastest-growing employment sectors (2), these low-wage, low-status jobs are difficult to fill and maintain with qualified people, especially in rural places (3). Personal assistance care in rural areas is burdensome both for disabled people and PCA workers: many people with self-care disabilities live in places where personal care attendants are in short supply (4) and the unpaid commuting “windshield time” required in rural areas limits worker availability and adds mileage costs (5). PCA positions rarely come with benefits and often require workers to combine several clients to reach full-time status and earnings (6). Many of the positions are also physically demanding and PCAs experience high rates of injury and disability (7). Finally, like many care work positions, the vast majority of these jobs are occupied by women, women of color, and immigrants (4), who often face more exploitation than other workers. Despite the intertwined relationships that exist between the disabled people needing services and the workers providing them, advocates for these groups have not historically worked together to fight for protections and rights for both groups. This paper is a brief introduction to how the movements for disability rights and workers' rights evolved over the twentieth century, with a narrow focus on relevant federal policies. We recognize that these programs are executed and managed by states and that state implementation is heterogeneous. Given the brief nature of this focused mini-review, we are unable to speak to these between-state differences or the conflicts that have arisen due to the complexities of implementing federal policies at the state level, sometimes without additional federal support.

The disagreement regarding what policies are needed is embedded in the seemingly-competing goals of protecting both the “choice and control” (8) of disabled people and the labor policies needed to protect and promote workers' rights. On the side of protecting disabled people's autonomy, rural disability advocates recognized their unique needs were not always included in the dominant, urban-based movement for disability rights. This led to the formation of the Association of Programs for Rural Independent Living (9). However, there has been little organized support for rural PCAs. The goal of concurrently promoting and protecting both parties has historically been impeded by a belief among some disability rights advocates that if workers' rights and statuses are elevated, the autonomy of, and access to care for, people with disabilities will be demoted (10). To further understand the challenges of elevating and protecting both disabled people and workers, especially those living and working in rural areas, this paper (1) provides an overview of intersecting policies implemented since the 1930s, (2) considers the sources of supports and resistance in both movements, and (3) highlights the intersections of urbanormativity, ableism, and sexism in shaping policies and practices. This paper ends with a discussion of the current emerging opportunities in addressing the needs of both rural consumers and workers.

## Changing Policies Since the 1930s

While advocacy for disability rights has been formally happening for more than 150 years, advocacy at the national level for supports specific to being able to receive the care and services needed to stay in one's community and home have only come about more recently (11). The earliest policies focused on “protecting” non-disabled citizens from being exposed to people with disabilities (e.g., ugly laws). Many of these policies led to the hiding away of disabled individuals and kept them out of the labor market, with the exception of venues like “freak shows.” People with disabilities have been incredibly marginalized throughout history, including being primary targets of the Eugenics movement. It is estimated that 60,000 disabled people in the United States were subject to forced sterilization during this period; worldwide, the number is over a half of a million (12, 13). Slowly, US policies have evolved to support greater integration of disabled people into society. However, policy nuances have resulted in less progress for disabled people in rural places. For example, employment provisions within the Americans with Disabilities Act (14) applied only to businesses that employed more than 15 people. Given employers in rural areas tend to be small businesses with fewer than 15 employees, rural disabled people benefit less from this policy than their urban counterparts (15).

Table 1 is a very brief overview of some of the key federal-policy-related events that impacted both the evolution of policies related to personal assistance services and home care workers' rights. For both people with disabilities and PCAs, the federal government's response to the Great Depression was a turning point, bringing some of the inequities and challenges faced by both groups to light. The Social Security Act (16) established formal federal funding (distributed to the states) for supporting people with disabilities, primarily in institutional or group living situations (10). Similarly, under the Roosevelt administration, the Visiting Housekeeping Program was established as part of The New Deal (17). This program put women, including many women of color, to work in other people's homes. Training centers for these programs were primarily located in urban centers, likely drawing labor-seeking women from the countryside. Despite the important gains made in passing the Fair Labor Standards Act of 1938 to protect workers from the most harsh and unsafe working conditions and to limit the standard work week, 86% of working women, including PCAs, were not included in the protections (17).

**Table 1:** Evolution of federal policies related to personal assistance services and home care workers' rights.

**Home and community-based services**	**Home care workers' rights**
•1935—The Social Security Act established formal funding streams for supports for people with disabilities, primarily in institutional or group home settings^a^. • 1950—Social Security Act Amendment mandated Medicaid payment go directly to nursing homes, rather than beneficiary^a^. • 1956—Social Security Disability Insurance established to support low-income disabled people^a^. • 1961—Community Health Services and Facilities Act^a^. • 1962—President Kennedy formed a President's Panel to address federal policies for people with intellectual disabilities, including the need for workers to support and provide care for these people^b^. • 1963—President Kennedy asks Congress to address the mass institutionalization of people with disabilities^a^ and signed into law an act that created a national network of University Centers for Excellence in Developmental Disabilities (UCEDDs), which support research, service, and training related to disability^b^. • 1970—Mandate for Medicaid to cover home-based care^a^. • 1973—The Rehabilitation Act prohibited discrimination against disabled people in the pursuit of employment and community participation by federally-funded entities and established nationwide centers for independent living^a^. • 1975—Social Security Act Amendment established first round of federal funding to incentivize states to move from institutional to home-based care^a^.	•1933—The Visiting Housekeeping Program was established as part of The New Deal^f^. • 1938—The Fair Labor Standards Act failed to include 86% of women, including home care workers, from protections pertaining to wages and work hours^f^. • 1961—Community Health Services and Facilities Act was passed, funding non-profit agencies to provide home-based PAS^f^. • 1964—Economic Opportunity Act authorized efforts to increase workers' wages^g^. • 1966—Economic Opportunity Amendment was created to fund training for those of low income to become trained home care paraprofessionals^g^. • 1967—Social Security Act Public Welfare Amendments were passed with a Worker Incentive Program to train housekeepers to aid older adults or individuals with disabilities. However, these jobs paid lower wages than did jobs for homemakers trained to aid in child care^h^. • 1974—Amendments were added to the Fair Labor Standards Act, providing wage and hour protections to domestic workers but not to home-based PAS workers due to a “companionship” exemption^i^.
•1990—The Americans with Disabilities Act guaranteed disabled people equal opportunities to employment, government services, and access to public buildings, including making modifications to avoid discrimination based on disability status^a^.	
•1993—PAS were formally included in Medicaid regulations. States were explicitly allowed to provide PAS outside of consumers homes^a^.	
•1999—The U.S. Supreme Court, in Omstead v. L.C., held that unjustified segregation of disabled people is unlawful discrimination under the Americans with Disabilities Act and that, under some conditions, public entities must provide HCBS to people with disabilities^a^.	
•1999—Medicaid Manual Transmittal authorized additional assistance with instrumental activities of daily living, such as transportation services, and authorized some types of family members to become paid providers of PAS^a^.	
•2001—The Real Choice Systems Change Grant Program was created through the Centers for Medicare and Medicaid Services to help states transform their long-term services and supports through awards to states to increase HCBS^a^.	
•2005—Safe, Accountable, Flexible, Efficient Transportation Equity Act: A Legacy for Users provides for investment in and development of accessible transportation in rural areas, with impacts on rural people with disabilities and their service providers^c^.	
•2005—The Deficit Reduction Act created the Money Follows the Person Program in support of state efforts to rebalance their LTSS systems by providing financial assistance to support increased use of HCBS and reduction of institutional living facilities^a^. • 2014—Medicaid HCBS Final Rule defines requirements for person-centered planning and adds protections for service recipients^d^. • 2016—Medicaid Managed Care Final Rule required states to identify people with LTSS needs and required managed LTSS plans to follow the requirements of Medicaid's person-centered service planning^e^.	•2014—The Harris v. Quinn court ruling held that homecare workers experienced a violation in their first amendment rights when forced to pay union dues^j^. • 2015—Fair Labor Standards Act “companion exclusion” was revised and protections were extended to home care workers^i^.

*This table includes major federal policies relevant to personal assistance services and home care workers' rights. It is not a comprehensive policy review and does not include state policies*.

*HCBS, home and community-based services; LTSS, long-term services and supports; PAS, personal assistance services*.

*^a^Nielsen (37)*.

*^b^Association of University Centers on Disabilities (38)*.

*^c^Yusuf and Mahar (39)*.

*^d^Centers for Medicare and Medicaid Services (40)*.

*^e^Paradise and Muscumeci (41)*.

*^f^Boris and Klein (17)*.

*^g^Nittoli and Giloth (42)*.

*^h^U.S. Senate Committee on Finance and U.S. House of Representatives Committee on Ways and Means (43)*.

*^i^U.S. Department of Labor, Employment Standards, Administration, Wage, and Hour Division (21)*.

*^j^U.S. Supreme Court (20)*.

After a period of national focus on war efforts following the New Deal policies, changes for disability rights picked up again in the 1950s, but policy implementation impacting the work of PCAs stayed fairly mute until the 1974 amendment to the Fair Labor Standards Act. This amendment explicitly excluded domestic workers (including PCAs) from protections, designating their work “companionship services.” During the 1950s, disability rights advocates gained ground in securing funding for basic living needs via Social Security Disability Insurance in 1956. Later advocacy by disability rights activists against institutionalization, and in favor of home-based services, resulted in amendments to the Social Security Act and new mandates throughout the 1960s and 1970s (see Table 1). During this time, however, there was little policy formation around the rights and working conditions of PCAs. Additionally, implementation of policies related to home-based services was slow, in part due to the growing power and influence of the nursing home industry (10). Though not perfect, formal programming and some fiscal supports were established during the 1960s and 1970s to meet federal mandates that Medicaid funding be used to support disabled people in their homes, rather than only in institutions. This would not become the Home and Community-Based Services program until 1983 when Congress added section 1915(c) to the Social Security Act (17), but these pieces of federal legislation and related policies provided important foundational support for today's systems.

From the 1990s to present, policy changes have led to substantial advances in conceptualizing disability and associated civil rights for disabled people (see Table 1), such as the Americans with Disabilities Act of 1990, having Personal Assistance Services formally included in Medicaid Regulations in 1993 (17), the Olmstead decision by the Supreme Court in 1999 (18), and the development and evaluation of the Money Follows the Person Program of 2005 (19). Policies in support of workers' rights have expanded to include First Amendment Rights protections for PCAs (20) and the 2015 removal of the 1974 companionship exception from the Fair Labor Standards Act (21). To follow is a brief discussion of some of the people, organizations, and industries involved in supporting and resisting changes for disabled people and PCAs.

## Sources of Support and Resistance

On the surface, home-based services for people with disabilities received public support. For instance, social reformers Reverend Louis Dwight and Dorothea Dix were among the first advocates to publicly criticize the deplorable living conditions of institutionalized individuals in the mid to late 1800s (22). As public consciousness about dignity of life for disabled people was elevated, it seems very few believed disabled people *should* be living in such conditions. It is notable that these institutions were largely operated in rural locations in the United States and hidden away from urban centers. These institutions provided jobs and economic support in many rural communities. However, this commodification of care for disabled residents attracted for-profit companies into the industry (15). The movement to deinstitutionalize disabled people did not really take hold until the 1950s, following the foundational policies established in the amendments to the Social Security Act (23). Societal events leading up to these changes included the widespread effects of polio outbreaks in 1916 and between 1949 and 1952 leading to higher rates of disability (24) and the presidential election of Franklin D. Roosevelt (who used a wheelchair), which helped shift the ways in which Americans thought about physical and mobility-related disabilities. Although deinstitutionalization of disabled people eradicated many residential institutions, nursing homes—which are also disproportionately concentrated in rural places—have in some ways taken their places (15).

The nursing home industry, with strong lobbying abilities, resisted home-based services (10) and won most of the policy battles, garnering Congressional support in amendments to the Social Security Act until the 1970s when it was mandated that nursing home-level care for people with disabilities on Medicaid must be covered in-home, if a disabled person chooses in-home care. However, the systems to accommodate these choices would be long in the making. The nursing home industry also played a role in the continued exclusion of home-based PCAs from federally protected workers' rights, arguing they could not afford to adhere to the protections for their institutional-based workers who were also excluded (17). Instead, PCAs in the US were subject to unjust working conditions such not being able to receive phone calls or spend time with friends if they lived with the person for whom they provided services and unclear limits on how many hours they were allowed or required to work (25). Additionally, international workers' rights were not protected to ensure a pathway to achieving immigration status, and they were instead faced with having to comply with their employer or risk deportation (25).

Home care worker unions such as the Service Employees International Union grew exponentially during the last 20 years. This led to many key protections for unionized workers in select states (26). However, supporters of home care workers' rights have experienced setbacks to their efforts to improve working conditions and wages in recent years. In 2018, the U.S. Supreme Court prohibited home care workers unions from charging non-members fees. The following year, in 2019, a Medicaid policy change barred home healthcare aides working for Medicaid-funded facilities and agencies from having union dues automatically deducted from their pay checks (27). The inability to more easily pay union dues has led to less union membership, fewer resources, and less collective bargaining power. Perhaps due to the incredible harsh and negative impacts of worker shortages during the COVID-19 pandemic (28), there has been recent momentum in disability rights advocates joining forces with workers' rights advocates to fight for better compensation and work conditions.

## Intersections of Urbanormativity, Ableism, and Sexism

With more awareness and support, the Independent Living Movement took hold in the mid-twentieth century and was intimately tied to other civil rights movements. With a mantra of “nothing about us without us” to acknowledge the long paternalistic history of making decisions about disabled bodies *for* people with disabilities rather than *with* them (26), disability rights advocates continue to fight for justice and equity today.

Like many other social justice events, disability advocacy has largely taken place in urban areas [e.g., (28)]. With the exception of work done by the fairly small organization, the Association of Programs for Rural Independent Living, the Independent Living Movement has been fairly urbancentric with most activity happening on university campuses and in cities (8), making it difficult for rural disabled people to participate.

Given the urban focus of the Independent Living Movement, it is perhaps unsurprising rural-specific issues related to receiving personal assistance services have neither been sufficiently addressed nor researched thoroughly. Furthermore, in rural places, lack of affordable and accessible housing and limited availability of PCAs has led to unjust institutionalization of disabled people in nursing homes (15). Next, we briefly explore how ableism and sexism have played a role in the evolution of these policies influencing rural care work and those who need services.

From the beginning, there has been resistance to financially supporting people with disabilities at adequate levels. Some of this resistance is embedded in a cultural belief in rugged independence and self-sufficiency, which is more prevalent among rural citizens (11). Our country has a long history of having a weak safety net that is slow to kick in and quick to be pulled back (29). The evolving medical field and technology provided decision makers with new tools to determine who was “deserving” and “undeserving” of community living and services, as evidenced by the strict and extremely complex protocols established to determine eligibility for Social Security Disability Insurance (10). All of this, in addition to employment-based health benefits, contributes to keeping workers tied to the labor market.

The Visiting Housekeeping Program served as a catalyst for propelling PCAs toward a more formalized and professionalized occupation. However, it was met with resistance from the Southern textiles and manufacturing industry leaders because they argued that as they were getting back on their feet, they could not compete with subsidized wages provided by the government (17). This intersected with the restriction that only one person per family could be supported by Worker Progress Administration programs (which included the Visiting Housekeeping Program), which favored men (17). Finally, because care in the home was seen as less valuable than other labor, it was difficult for workers' rights advocates to gain any momentum toward better compensation and work conditions. This particular belief also helped fuel the resistance to workers' rights among people with disabilities who desired high degrees of autonomy and control in organizing their daily lives and services (30).

In terms of workers' rights, women in families with individuals with disabilities were historically and continue to be expected to provide family care for free, saving the government billions of dollars (31). In fact, currently 80% of care provided to people with disabilities and older adults is unpaid. Despite the majority of women being in the workforce by the late 1970s, family caregiving continues to be a social expectation, placing incredible burdens on many women (32). Even after the advent of Home and Community-Based Services, many states did not allow spouses or parents to be paid for providing care (33). These types of rules made it extremely hard for rural people needing services to find workers in their communities (4). However, today there is more momentum for creating better supports than has been seen for many, many years.

## Discussion

This paper highlights the complex social justice issues that arise when trying to elevate the needs of different groups that, at first, appear to have competing goals. This becomes even more complicated when we turn our attention toward the implications in rural places. The gains made by people with disabilities to have services that enable them to live, work, and recreate in community necessitate the commodification of other people's labor. In some cases, this means the autonomy of disabled people appears to be in conflict with the autonomy of workers, a conflict that is subsumed by a system that does not adequately support either group. For rural people with disabilities, current policies do not address the additional burden of rurality, including a lack of local workers (especially when spouses or parents are excluded from being paid caregivers), additional costs related to the lack of accessible, public transportation (34). For the workers who provide these essential services, workers' rights advocacy also has not addressed the additional burdens of “windshield time,” car maintenance, and the costs of providing care in less accessible homes and communities with fewer services, for lower wages compared to what they can earn providing care in urban places (35).

Based on this review and the growing interest in finding ways to better support both people with disabilities and PCAs, we recommend organizations doing research in home-based services—such as the AARP Public Policy Institute—consider adding rural components to their very useful Long-Term Services and Supports Scorecard analyses (36). Topics to consider include adjustment of wages to better compensate rural workers, better compensation for vehicles and mileage, and incentivizing individuals in rural places to become PCAs. It is also recommended these organizations employ staff knowledgeable in the unique history of, and issues faced by, rural disabled people and service providers. We also recommend including rural voices of people with disabilities and PCAs in relevant policy discussions and decisions. Finally, in advocacy work, we encourage social justice advocates to consider making room at the table for rural people impacted by these issues in ways that do not exacerbate the burden of participation faced by many rural people (e.g., driving long distances to participate in advocacy events).

## Author Contributions

Idea for the paper was by RS. Writing, critical review of the paper, suggested edits, revisions, and responses to reviewers were distributed evenly between RS, KS, and GM. Final edits were done by KS and GM. All authors contributed to the article and approved the resubmitted version.

## Funding

This work was supported by the Research and Training Center on Disability in Rural Communities (RTC:Rural) under a grant from the National Institute on Disability, Independent Living, and Rehabilitation Research (NIDILRR; grant number 90RTCP0002), which was a Center within the Administration for Community Living (ACL), Department of Health and Human Services (HHS).

## Author Disclaimer

The research does not necessarily represent the policy of NIDILRR, ACL, or HHS and one should not assume endorsement by the federal government.

## Conflict of Interest

The authors declare that the research was conducted in the absence of any commercial or financial relationships that could be construed as a potential conflict of interest.

## Publisher's Note

All claims expressed in this article are solely those of the authors and do not necessarily represent those of their affiliated organizations, or those of the publisher, the editors and the reviewers. Any product that may be evaluated in this article, or claim that may be made by its manufacturer, is not guaranteed or endorsed by the publisher.

## Footnotes

^1^The terms disabled people (identity first language) and people with disabilities (person first language) are used interchangeably to reflect the current preferences of advocates in the disability rights field.
